# Launching Plasmons in a Two-Dimensional Material Traversed by a Fast Charged Particle

**DOI:** 10.3390/ma16031150

**Published:** 2023-01-29

**Authors:** Gareth Arturo Marks, Devin Blankespoor, Zoran L. Miskovic

**Affiliations:** 1 Department of Physics and Astronomy, University of Waterloo, Waterloo, ON N2L 3G1, Canada; 2 Department of Applied Mathematics, University of Waterloo, Waterloo, ON N2L 3G1, Canada; 3 Waterloo Institute for Nanotechnology, University of Waterloo, Waterloo, ON N2L 3G1, Canada

**Keywords:** plasmon, two-dimensional, image force, dielectric function, moving charge

## Abstract

We use a dielectric-response formalism to compute the induced charge density and the induced potential in a conductive two-dimensional (2D) material, traversed by a charged particle that moves on a perpendicular trajectory with constant velocity. By analyzing the electric force on the material via the Maxwell stress tensor, we showed that the polarization of the material can be decomposed into a conservative part related to the dynamic image force, and a dissipative part describing the energy and momentum transfer to the material, which is ultimately responsible for launching the plasma oscillation waves in the material. After showing that the launching dynamics is fully determined by the Loss function of the material, we used a conductivity model suitable for the terahertz to the midinfrared frequency range, which includes both the intraband and interband electron transitions in the material, to compute the real-space and time animations of the propagating plasma waves in the plane of the material. Finally, we used a stationary phase analysis to show that the plasmon wave crests go into an overdamped regime at large propagation distances, which are comparable to the distances where retardation effects are expected to emerge due to hybridization of the plasmon dispersion with the light line at long wavelengths.

## 1. Introduction

Due to its many interesting and potentially useful electrical properties, graphene, the archetypal example of a two-dimensional (2D) material, has received significant recent attention. Plasmonics of graphene-based heterostructures has become a well established research area [1,2,3,4,5,6,7], while new trends in the exploration of the light interaction with 2D nanomaterials continue to use graphene as a test system [8]. Some of this research endeavor has focused on the study of mechanisms leading to the plasmon excitation [9]. While the optical means of plasmon excitation in 2D materials require breaking their translational invariance by suitable patterning of their atomic lattice [9], the interaction with external localized probes, such as nanotips [10] or electron beams [11], provides conditions for the momentum matching with the plasmon wavevectors, which is necessary for launching the plasma waves. Those waves can then propagate over large distances across the uninterrupted surface of a 2D material if the plasmon damping processes are not too strong [12].

There has been recent interest in the interaction between fast charged particles and sheets of graphene, along with other 2D materials in the context of Electron Energy Loss Spectroscopy (EELS) in a Scanning Transmission Electron Microscope (STEM) [13,14,15,16]. This is a microscopy technique used in nanophotonics research to understand, among other properties, the nature of plasmon excitations in nanostructures by probing the energy and momentum transfer from a fast incident electron to the target material [17]. It is noteworthy that graphene and other 2D materials can be suspended over a holey grid in STEM-EELS and hence may be probed free from the influence of the nearby materials [18]. Such setting is ideally suited for realizing “splashing transients” in the plasmon waves excited in a 2D material by an impact of a fast electron [11], which can be assumed to traverse that material while experiencing negligible recoil. Theoretical investigation of the dynamics of the plasmon wave propagation can shed light on fundamental aspects of EELS in 2D materials, especially in the range of low energy losses, going down to the mid-infrared (MIR) range of frequencies [19]. This dynamics is governed by the dispersion relation between the plasmon eigenfrequency $\omega_{p}$ and the in-plane wavevector k, and the corresponding plasmon group velocity, $v_{p}=\nabla_{k}\omega_{p}$ [20,21].

In STEM and related methods, much attention has already been paid to the transfer of electromagnetic energy and momentum between fast electron beams and plasmonic nanoparticles. More pertinent to our ends, prior research has established, in the fully relativistic regime, the theory surrounding the energy loss channels for fast charged particles interacting with single or multiple layered sheets of graphene in a number of different configurations [22,23,24]. This is done by solving the macroscopic Maxwell’s equations, treating the graphene as a conducting planar sheet of zero thickness. Graphene in these models is thus modeled as containing a 2D free electron gas whose dynamic polarization is described by an in-plane conductivity σ. The choice of σ is thus the starting point for the modeling of plasmon excitations in the material, and it can be taken either from certain simple established models or obtained numerically from quantum-mechanical ab initio calculations [1,2,3,4,5,6,7]. This approach allows for easy handling of the system in the framework of the macroscopic Maxwell’s equations by use of familiar electromagnetic boundary conditions, supplemented by a constitutive relation that one can derive from the analysis of an in-plane Ohm’s law.

We limit our focus in this work on conductivity models capable of describing the so-called sheet plasmons in 2D materials that occur in the terahertz (THz) to the MIR range and may be defined as the collective modes of quasifree charge carriers in a material exhibiting metallic character, such as doped graphene [1], monolayer transition metal dichalcogenides (TMDs) [25], or doped 2D semiconductors like monolayer black phosphorus (phosphorene) [26]. These modes are governed at the quantum-mechanical level by the low-energy intraband electronic transitions in those materials, which are well described by a Drude model for their in-plane electrical conductivity in the limit of vanishing wavenumber. This model gives rise to the characteristic square-root dispersion of the plasmon frequency in 2D materials, $\omega_{p}\propto \sqrt{k}$, for small k=|k| values. However, it is also necessary to take into account a correction to the in-plane conductivity in the THz–MIR range, which comes from high-energy interband electron transitions in those materials [27,28,29]. It was shown that this correction gives rise to a saturation of the plasmon dispersion as the wavenumber *k* increases, resulting in slowing down of such plasmons [25,28,29]. Hence, it is worthwhile to explore the effects of this slowing down in the dynamics of launching such sheet plasmons by an impact of the incident charged particle.

**Figure 1:**
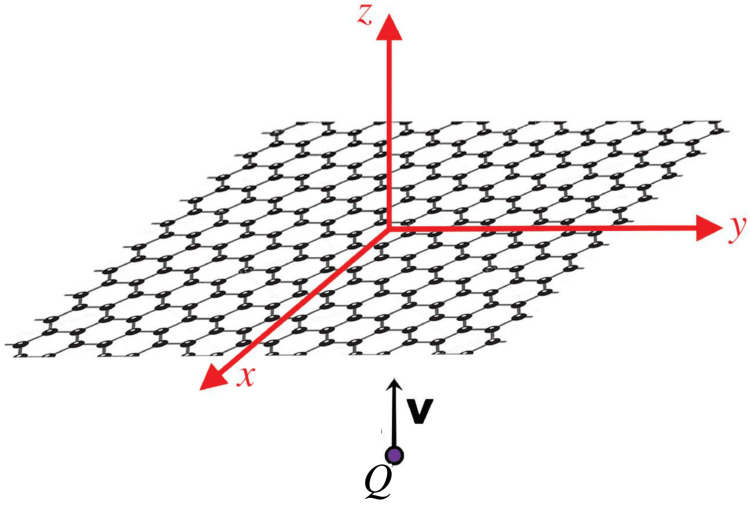
Schematic showing the situation we consider in this work, with the point charge *Q* approaching the graphene sheet on a normal trajectory (which we take to be along the *z*-axis) at constant velocity v.

The situation we wish to model in this report is shown in Figure 1. We take an infinite graphene sheet of zero thickness corresponding to the plane z=0, with a particle of charge *Q* moving down the *z*-axis at speed *v* so that it reaches the origin at time t=0. Note that the negligible deflection assumption imposes that *v* is sufficiently large that reaction forces exerted on it by the sheet disturb its trajectory only negligibly, as is typically the case in STEM experiments. In most practical applications, the charged particle will of course be an electron, but we will leave its charge arbitrary for full generality. Furthermore, while expressions for the fields are known in the fully relativistic regime, we will restrict our attention to the nonrelativistic approximation, leaving the general case to further study. This report will focus on understanding the nature of plasmon excitations in the sheet by studying the dispersion relation, surface charge distribution, surface electrostatic potential, and total forces exerted on the sheet for a chosen conductivity model. We will also show how it is possible to decompose the quantities relevant to total energy and momentum into a conservative and a dissipative part, even without choosing a conductivity model. While the conservative part is related to the dynamic image force on the moving charge, it is the dissipative part that is of physical interest in EELS because it is the only one contributing to the total energy/momentum transfer. We therefore identify it with the energy and momentum carried away by the plasmon waves excited inside a 2D material. Finally, we will show that the spatiotemporal dependencies of the associated charge density and electric potential in the 2D material are fully determined by the so-called Loss function, expressed in terms of an effective 2D dielectric permittivity of the material [30].

Unless stated otherwise, we shall use the Gaussian units of electrostatics.

## 2. Theoretical Model

### 2.1. Conventions for Fourier Transforms

Given some function *g* that depends on space coordinates (r,z) and time *t*, there are three representations we will consider, denoted as follows:

1. The purely spatiotemporal domain, denoted g(r,z,t) where $r=x\hat{x}+y\hat{y}$ is the radial vector parallel to the sheet, and *z* the axis perpendicular to it, see Figure 1.
2. The (k,t) domain with the *x* and *y* dependence Fourier transformed, where $k=k_{y}\hat{x}+k_{y}\hat{y}$, which we’ll denote with a caron accent, the transformation being according to the convention:
  (1) $$\overset{ˇ}{g}\left(k,z,t\right)=\;\int \int \;d^{2}r\;e^{-ik\cdot r}g\left(r,z,t\right),$$
  where $d^{2}r=dx\;dy$.
3. The (k,ω) domain in which time is also transformed into the frequency domain, which we’ll denote by a tilde:
  (2) $$\tilde{g}\left(k,z,\omega \right)=\int_{-\infty }^{\infty }dt\;e^{i\omega t}\overset{ˇ}{g}\left(k,z,t\right).$$

These conventions will be used throughout to indicate which domain we are working in. One reason for introducing these integral transformations is that it is often easier to solve Maxwell’s equations in a transformed domain than in the purely spatiotemporal one. For instance, and of particular importance for our purposes, the problem of solving for the fields for a charged particle penetrating a graphene sheet on a normal trajectory has been solved in the (k,ω) domain in a manner independent of the choice of conductivity model [22]. However, it is not possible to obtain analytic expressions in the spatiotemporal domain by performing the inverse Fourier transforms. Our analysis in the nonrelativistic regime will begin with the application of the methods described above used to study Poisson’s equation in the (k,ω) domain.

### 2.2. Poisson Equation in (k,ω) Domain

In the nonrelativistic approximation, all magnetic fields vanish, and the problem is most simply characterized by the scalar potential ϕ giving the electric field E by E=−∇ϕ. This approximation follows from the general expression for that field, $E=-\nabla \phi -c^{-1}\partial A/\partial t$ [31], where the chain rule can be used in the second term upon changing the time variable to t=z/v for a point charge that moves along the *z* axis at constant speed v>0. Then, the second term with the vector potential A may be neglected by assuming v≪c, where *c* is the speed of light. While this assumption is necessary to validate the neglect of the magnetic fields due to the moving particle, a fully relativistic formulation of the problem showed that the magnetic field due to the induced current in a conducting sheet may only be neglected if one *additionally* assumes that ω≪kc, see Equation (6) in [17]. This inequality is automatically satisfied by assuming v≪c for a particle moving parallel to the sheet because of the resonance condition ω=kv [28,29], but for the perpendicular trajectory in Figure 1, a strong hybridization of the plasmon dispersion with the light line ω=ck can affect the launching of plasmons with long wavelengths, even when v≪c. The proximity of the region ω>ck, which was shown to give rise to a sizeable radiative energy loss in doped graphene in the THz-MIR frequency range [22,23,24], renders the issue of the nonrelativistic approximation rather nontrivial in the present context. Nevertheless, we expect that our results will be applicable for the incident electron energies in the low–keV range, and may be of a semiquantitative utility for typical incident energies in the monochromated STEM-EELS with ultrahigh energy resolution [19,32].

We furthermore associate with the graphene sheet the surface charge density $\rho_{2D}\left(r,t\right)$, induced by a moving external charge. These quantities are related in the Fourier domain by a constitutive relation

(3) $${\tilde{\rho }}_{2D}\left(k,\omega \right)=-e^{2}\chi \left(k,\omega \right)\tilde{\phi }\left(k,0,\omega \right),$$

where χ(k,t) is the density–density polarization function of noninteracting electrons [33], related to the in-plane conductivity of the material σ by

(4) $$\chi \left(k,\omega \right)=\frac{ik^{2}}{e^{2}\omega }\sigma \left(k,\omega \right),$$

which can be obtained by the analysis of an in-plane Ohm’s law and the continuity equation for the charge density in the material. We now wish to solve the Poisson’s equation $\nabla^{2}\phi =4\pi \rho$ in the presence of a point charge *Q*, moving along the *z* axis at constant speed *v*, see Figure 1. In the (k,ω) domain, this can be written as

(5) $$\frac{\partial^{2}\tilde{\phi }}{\partial z^{2}}-k^{2}\tilde{\phi }=-4\pi \left[{\tilde{\rho }}_{\mathrm{ext}}\left(k,z,\omega \right)+\delta \left(z\right){\tilde{\rho }}_{2D}\left(k,\omega \right)\right]$$

where

(6) $${\tilde{\rho }}_{\mathrm{ext}}\left(k,z,\omega \right)=Q\;e^{i\omega z/v}$$

is the external charge density due to the point charge itself, which follows from Fourier transforming $\rho_{\mathrm{ext}}\left(r,z,t\right)=Q\delta \left(z-vt\right)\delta^{2}\left(r\right)$. We may furthermore express the total potential ϕ as the sum of an external and induced part,

(7) $$\phi =\phi_{\mathrm{ext}}+\phi_{\mathrm{ind}},$$

where $\phi_{\mathrm{ext}}$ is merely the Coulomb potential due to the moving point charge,

(8) $$\phi_{\mathrm{ext}}\left(r,z,t\right)=\frac{Q}{{\left(r^{2}+{\left(z-vt\right)}^{2}\right)}^{1/2}},$$

which gives

(9) $${\overset{ˇ}{\phi }}_{\mathrm{ext}}\left(k,z,t\right)=\frac{2\pi }{k}Q\;e^{-k|z-vt|},$$

and

(10) $${\tilde{\phi }}_{\mathrm{ext}}\left(k,z,\omega \right)=\frac{4\pi Qv}{\omega^{2}+{\left(kv\right)}^{2}}\;e^{i\omega z/v},$$

whereas the induced part solves

(11) $$\frac{\partial^{2}{\tilde{\phi }}_{\mathrm{ind}}}{\partial z^{2}}-k^{2}{\tilde{\phi }}_{\mathrm{ind}}=-4\pi \delta \left(z\right){\tilde{\rho }}_{2D}\left(k,\omega \right).$$

This equation may be solved in piecewise manner for z≠0 by applying the familiar boundary/matching condition [31],

(12) $$\frac{\partial {\tilde{\phi }}_{\mathrm{ind}}}{\partial z}{|}_{z=0^{+}}-\frac{\partial {\tilde{\phi }}_{\mathrm{ind}}}{\partial z}{|}_{z=0^{-}}=-4\pi {\tilde{\rho }}_{2D}\left(k,\omega \right),$$

where we have taken into account that the derivative $\frac{\partial }{\partial z}{\tilde{\phi }}_{\mathrm{ext}}\left(k,z,\omega \right)$ from (10) is a continuous function in the neighborhood of z=0. Upon using the constitutive relation (3) in the right-hand side of (12), we finally obtain

(13) $${\tilde{\phi }}_{\mathrm{ind}}\left(k,z,\omega \right)=\left[\frac{1}{\epsilon_{2D}\left(k,\omega \right)}-1\right]{\tilde{\phi }}_{\mathrm{ext}}\left(k,0,\omega \right)\;e^{-k|z|},$$

where we have introduced an effective 2D dielectric permittivity in the plane z=0,

(14) $$\epsilon_{2D}\left(k,\omega \right)=1+2\pi i\frac{k}{\omega }\sigma \left(k,\omega \right),$$

where we used (4) to express the permittivity of a 2D material in terms of its in-plane conductivity, which is what we will choose models for. Notice that we then have

(15) $$\tilde{\phi }\left(k,0,\omega \right)=\frac{{\tilde{\phi }}_{\mathrm{ext}}\left(k,0,\omega \right)}{\epsilon_{2D}\left(k,\omega \right)}$$

showing the origin of the identification of $\epsilon_{2D}$ as an effective permittivity of the 2D material, as it rescales the in-plane external potential in the (k,ω) domain to give the total potential.

Obtaining numerical results in the spatiotemporal domain for the induced potential in the plane z=0, $\phi_{\mathrm{ind}}\left(r,0,t\right)$, and the induced surface charge density, $\rho_{2D}\left(r,t\right)$, is one of the main aims of this report. This will be achieved by first performing analytically the inverse Fourier transform with respect to frequency using a suitable model of the conductivity in the expressions

(16) $${\tilde{\phi }}_{\mathrm{ind}}\left(k,0,\omega \right)=\frac{4\pi Qv}{\omega^{2}+{\left(kv\right)}^{2}}\left[\frac{1}{\epsilon_{2D}\left(k,\omega \right)}-1\right]$$

and

(17) $${\tilde{\rho }}_{2D}\left(k,\omega \right)=\frac{k}{2\pi }{\tilde{\phi }}_{\mathrm{ind}}\left(k,0,\omega \right),$$

which follows from (12) and (13). Of course, once we have these, the boundary condition (12) allows us to obtain the force densities exerted on the sheet, allowing us to compute the energy and momentum transfer if desired.

### 2.3. Separation of Conservative and Dissipative Processes for Arbitrary Conductivity

Even before we commit to the study of a particular conductivity model, we can make a number of theoretical developments assuming that the 2D sheet is isotropic, so that its conductivity depends on the norm k=|k| and is also an arbitrary complex function of frequency ω that must satisfy certain general properties [22,28]. The quantity of primary interest in this section is the integral

(18) $$I\left(k,t\right)=\int_{-\infty }^{\infty }d\omega \frac{e^{-i\omega t}}{\omega^{2}+{\left(kv\right)}^{2}}\left[1-\frac{1}{\epsilon_{2D}\left(k,\omega \right)}\right]$$

which is related to the expression for the induced potential in the (k,t)-domain by

(19) $${\overset{ˇ}{\phi }}_{\mathrm{ind}}\left(k,0,t\right)=-2QvI\left(k,t\right),$$

as can be seen from our convention (2) for the Fourier transform. Later we will see that this integral can be carried out analytically for certain common choices of σ. At this stage, however, we will find it useful to decompose this integral into a conservative and dissipative part, the latter beginning to contribute at t=0:

(20) $$I\left(k,t\right)=I_{\mathrm{cons}}\left(k,t\right)+H\left(t\right)I_{\mathrm{diss}}\left(k,t\right),$$

where H(t) is the Heaviside step function, and

(21) $$I_{\mathrm{cons}}\left(k,t\right)=\frac{\pi }{kv}e^{-kv|t|}\left[1-\frac{1}{\epsilon_{2D}\left(k,ikv\right)}\right],$$

(22) Idiss(k,t)=4∫0∞dωsin(ωt)ω2+(kv)2Im−1ϵ2D(k,ω).

The decomposition in (20) with (21) and (22) generalizes a similar approach adopted in [20] to arbitrary dielectric permittivity. As will be shown below, the key property of $I_{\mathrm{cons}}\left(k,t\right)$ that makes it conservative is that it is symmetric (even) function of *t*. On the other hand, the dissipative part $I_{\mathrm{diss}}\left(k,t\right)$ will be shown below to be responsible for launching plasmon waves in the 2D material upon the impact of the charged particle, which were found in Refs. [11,20] to arise only at times t>0, i.e., after the particle passes through the material.

The decomposition in (20) is based on the following considerations when applying the residue theorem to the integral in (18) [34]. This is done by extending the integrand to a function of complex-valued ω and recalling that $\epsilon_{2D}$ can always be considered as a function R×C→C satisfying [35]

(23) $$\epsilon_{2D}\left(k,-\omega^{*}\right)=\epsilon_{2D}^{*}\left(k,\omega \right),$$

where ∗ indicates complex conjugate. Moreover, in order to satisfy the causality principle, a realistic response function $R\left(k,\omega \right)=1/\epsilon_{2D}\left(k,\omega \right)$, or the generalized susceptibility of the 2D material that appears in (15), must be regular in the upper half of the complex-ω plane and cannot have poles along the Re(ω) axis [35]. At the same time, the kinematic factor in the integrand of (18) has simple poles at ω=±ikv, which may be taken into account by integrating along the Re(ω) axis while closing the contour with a semicircle of radius approaching infinity that is placed in the upper half-plane for t<0 and in the lower half-plane for t>0. However, the conservative term in (20) originates from the pole at ikv in the upper half-plane (without loss of generality, we may assume that the particle moves in the positive direction of the *z* axis, so that v>0). The contribution from that pole to the contour integrals arising from (18) for both positive and negative times can be separated out by using the identity [34]

(24) $$e^{-i\omega t}=\left\{\begin{array}{cc} e^{-i\omega t},\; & t<0,\\ e^{i\omega t}-2i\sin\left(\omega t\right),\; & t>0. \end{array}\right.$$

Namely, the terms with $e^{\mp i\omega t}$ in the right-hand side of (24) can be integrated by closing the contour in the upper half plane for both t<0 and t>0, giving (21), whereas the term sin(ωt) only “kicks in” for t>0 and may be integrated over the Re(ω) axis, which gives (22) upon using the property (23).

The origin of the naming conventions in (21) and (22) is as follows. It will be shown below that the part of the induced potential due to $I_{\mathrm{cons}}$, which generally dominates in magnitude, contributes nothing to the overall energy/momentum transfer between the electron and the sheet. In fact, it may be traced to the (dynamic) image potential that results from an elastic polarization of the electron system in the material, which is described by the appearance of the dielectric permittivity $\epsilon_{2D}\left(k,\omega \right)$ at the “imaginary” frequency ω=ikv in (21). One may recall that the response function $R\left(k,\omega \right)=1/\epsilon_{2D}\left(k,\omega \right)$, is generally real-valued along the positive part of the imaginary frequency axis, where it is a monotonically decreasing function of Im(ω) [35]. On the other hand, the dissipative part $I_{\mathrm{diss}}$ is the term of physical interest in the EELS. The quantity Im−1ϵ2D(k,ω) that appears in (22) is the so-called Loss function, providing a quantitative measure of the tendency of the material to absorb electromagnetic energy and momentum at each point in (k,ω)-space [30]. It accounts for the entirety of the energy/momentum transfer, which may go directly into single-particle excitations in the material, or to the excitation of collective modes of oscillation, such as a plasma wave, which will ultimately dissipate their energy into the Joule heat in the material. If the material supports a long-lived collective mode, like a plasmon with dispersion relation $\omega =\omega_{p}\left(k\right)$ in the idealized limit of negligible damping, then the loss function may be written in terms of a delta function as Im−1ϵ2D(k,ω)=A(k)δ(ω−ωp(k)). Using this form of the loss function in (22) indicates that it would be more appropriate, in this idealized case, to call $I_{\mathrm{diss}}\left(k,t\right)$ a “plasmonic” part.

Once a simple enough model is chosen for σ(k,ω), which yields an analytical expression for $I_{\mathrm{diss}}\left(k,t\right)$, the resulting I(k,t) from (20) can be used along with (19) to perform an inverse Fourier transform pertaining to (1). As a consequence, the spatiotemporal dependence of the axially symmetric induced potential can be calculated by performing a single integration according to

(25) $$\phi_{\mathrm{ind}}\left(r,0,t\right)=-\frac{Qv}{\pi }\int_{0}^{\infty }dk\;k\;J_{0}\left(kr\right)\;I\left(k,t\right),$$

where $J_{0}$ is a Bessel function, with a similar integral for the induced charge density,

(26) $$\rho_{2D}\left(r,t\right)=-\frac{Qv}{2\pi^{2}}\int_{0}^{\infty }dk\;k^{2}\;J_{0}\left(kr\right)\;I\left(k,t\right).$$

We may conclude that the decomposition in (20) gives rise to the corresponding decompositions of both the induced potential and the induced density as

(27) $$\phi_{\mathrm{ind}}\left(r,0,t\right)=\phi_{\mathrm{cons}}\left(r,0,t\right)+\phi_{\mathrm{diss}}\left(r,0,t\right),$$

(28) $$\rho_{2D}\left(r,t\right)=\rho_{\mathrm{cons}}\left(r,t\right)+\rho_{\mathrm{diss}}\left(r,t\right),$$

the computations of which will constitute the main results of this paper.

### 2.4. Forces and Energy-Momentum Conservation

To further investigate conservative and dissipative interactions, we derive expression for the total force acting on the 2D material using the approach of [17] based on the Maxwell stress tensor formalism. By placing the material in the region Ω defined by a slice −d<z<d where *d* is allowed to approach zero, we may express the force as the flux of the Maxwell stress tensor $\overset{↔}{T}$

(29) $$F\left(t\right)={\;\int \int \;}_{\partial \Omega }\overset{↔}{T}\left(r,z,t\right)\cdot \hat{n}\;dA,$$

where the surface ∂Ω consists of two planes normal to the *z*-axis and infinitesimally above and below z=0, respectively. The entries of the Maxwell stress tensor are given in the nonretarded limit in terms of the electric field components by [31]

(30) $$T_{ij}=\frac{1}{4\pi }\left(E_{i}E_{j}-\frac{1}{2}E^{2}\delta_{ij}\right).$$

It can be shown that the tangential components of the total force in (29) on the 2D material vanish by symmetry, while the perpendicular force becomes

(31) $$F_{z}\left(t\right)=\;\int \int \;d^{2}r\;\left[T_{zz}\left(r,0^{+},t\right)-T_{zz}\left(r,0^{-},t\right)\right].$$

The symmetry of the problem is best handled by switching from a double integral in (31) over the (x,y) domain to a double integral in the $\left(k_{x},k_{y}\right)$ domain by using a generalization of Parseval’s theorem, which yields for typical contributing terms to the total force

(32) $$\begin{array}{c} \;\int \int \;d^{2}r\;E_{i}\left(r,z,t\right)E_{j}\left(r,z,t\right){|}_{z=0^{-}}^{z=0^{+}}=\;\int \int \;\frac{d^{2}k}{{\left(2\pi \right)}^{2}}\;{\overset{ˇ}{E}}_{i}\left(k,z,t\right){\overset{ˇ}{E}}_{j}\left(-k,z,t\right){|}_{z=0^{-}}^{z=0^{+}}, \end{array}$$

where $d^{2}k=dk_{x}\;dk_{y}$. Recall that ${\overset{ˇ}{E}}_{j}\left(-k,z,t\right)={\overset{ˇ}{E}}_{j}^{*}\left(k,z,t\right)$ in the above formula. Furthermore, the field components tangential to the plane z=0 are continuous across the plane, and therefore make no contribution when integrated over in (31). Therefore, we only need to evaluate the force perpendicular to the 2D material,

(33) $$F_{z}\left(t\right)=\frac{1}{8\pi }\;\int \int \;\frac{d^{2}k}{{\left(2\pi \right)}^{2}}\;{\overset{ˇ}{E}}_{z}\left(k,z,t\right){\overset{ˇ}{E}}_{z}\left(-k,z,t\right){|}_{z=0^{-}}^{z=0^{+}},$$

where the *z*-component of the electric field may be written using (7) as ${\overset{ˇ}{E}}_{z}\left(k,z,t\right)={\overset{ˇ}{E}}_{z}^{\mathrm{ext}}\left(k,z,t\right)+{\overset{ˇ}{E}}_{z}^{\mathrm{ind}}\left(k,z,t\right)$. Referring to (9), we see that ${\overset{ˇ}{E}}_{z}^{\mathrm{ext}}\left(k,z,t\right)=-\frac{\partial }{\partial z}{\overset{ˇ}{\phi }}_{\mathrm{ext}}\left(k,z,t\right)$ is a continuous function in the neighborhood of z=0 (except when t=0), which takes the in-plane value given by

(34) $${\overset{ˇ}{E}}_{z}^{\mathrm{ext}}\left(k,0,t\right)=-2\pi Q\;sign\left(t\right)\;e^{-kv|t|},$$

where sign is a signum function. On the other hand, referring to (13), one may infer that ${\overset{ˇ}{E}}_{z}^{\mathrm{ind}}\left(k,z,t\right)=-\frac{\partial }{\partial z}{\overset{ˇ}{\phi }}_{\mathrm{ind}}\left(k,z,t\right)$ has a jump at z=0, which accounts for the left-hand side of (12), written in the (k,t) domain as

(35) $${\overset{ˇ}{E}}_{z}^{\mathrm{ind}}\left(k,0^{+},t\right)-{\overset{ˇ}{E}}_{z}^{\mathrm{ind}}\left(k,0^{-},t\right)=4\pi {\overset{ˇ}{\rho }}_{2D}\left(k,t\right).$$

Therefore, by inserting ${\overset{ˇ}{E}}_{z}={\overset{ˇ}{E}}_{z}^{\mathrm{ext}}+{\overset{ˇ}{E}}_{z}^{\mathrm{ind}}$ in (33), we finally obtain

(36) $$\begin{array}{ccc} F_{z}\left(t\right) & = & \frac{1}{8\pi }\;\int \int \;\frac{d^{2}k}{{\left(2\pi \right)}^{2}}\;{\left.{\left|{\overset{ˇ}{E}}_{z}^{\mathrm{ext}}\left(k,z,t\right)+{\overset{ˇ}{E}}_{z}^{\mathrm{ind}}\left(k,z,t\right)\right|}^{2}\right|}_{z=0^{-}}^{z=0^{+}}\\ & = & \frac{1}{4\pi }\;\int \int \;\frac{d^{2}k}{{\left(2\pi \right)}^{2}}\;{\overset{ˇ}{E}}_{z}^{\mathrm{ext}}\left(-k,0,t\right)\left[{\overset{ˇ}{E}}_{z}^{\mathrm{ind}}\left(k,0^{+},t\right)-{\overset{ˇ}{E}}_{z}^{\mathrm{ind}}\left(k,0^{-},t\right)\right]\\ & = & \;\int \int \;\frac{d^{2}k}{{\left(2\pi \right)}^{2}}\;{\overset{ˇ}{E}}_{z}^{\mathrm{ext}}\left(-k,0,t\right){\overset{ˇ}{\rho }}_{2D}\left(k,t\right), \end{array}$$

where we used (35). The above result is consistent with a fully relativistic formulation of the force, see the first two lines of Equation (28) in [17].

When we insert in (36) the decomposition of the induced charge density ${\overset{ˇ}{\rho }}_{2D}\left(k,t\right)$ given in (28) and written in the (k,t) domain, we conclude from (36) that the perpendicular force on the 2D material may be analogously decomposed into a conservative part and a dissipative part,

(37) $$F_{z}\left(t\right)=F_{\mathrm{cons}}\left(t\right)+F_{\mathrm{diss}}\left(t\right).$$

Using ${\overset{ˇ}{\rho }}_{\mathrm{cons}}\left(k,t\right)=-\frac{k}{\pi }QvI_{\mathrm{cons}}\left(k,t\right)$ in place of ${\overset{ˇ}{\rho }}_{2D}\left(k,t\right)$ in (36) along with (21) and (34), we see that the conservative force on the 2D material can be expressed as the negative of the image force acting on the moving charged particle,

(38) $$F_{\mathrm{cons}}\left(t\right)={\left.\frac{dU_{\mathrm{im}}\left(z\right)}{dz}\right|}_{z=vt},$$

where its dynamic image potential is given by

(39) $$U_{\mathrm{im}}\left(z\right)=\frac{Q^{2}}{2}\int_{0}^{\infty }dk\;e^{-2k|z|}\;\left[\frac{1}{\epsilon_{2D}\left(k,ikv\right)}-1\right]$$

(recall that, without loss of generality, we assumed v>0). Clearly, the integral of $F_{\mathrm{cons}}\left(t\right)$ over −∞<t<∞ gives zero, i.e., the momentum transferred from the incident particle to the 2D material under the action of the conservative force vanishes [17]. The conservative nature of the dynamic image force was discussed in [36].

On the other hand, using ${\overset{ˇ}{\rho }}_{\mathrm{diss}}\left(k,t\right)=-\frac{k}{\pi }QvH\left(t\right)I_{\mathrm{diss}}\left(k,t\right)$ in place of ${\overset{ˇ}{\rho }}_{2D}\left(k,t\right)$ in (36) along with (22) and (34), we obtain the dissipative force as

(40) Fdiss(t)=8Q2vH(t)∫∫d2k(2π)2ke−kvt∫0∞dωsin(ωt)ω2+(kv)2Im−1ϵ2D(k,ω).

We may integrate this expression over time [notice the Heaviside function H(t)] to find a momentum $p_{\mathrm{diss}}$ transferred to the 2D material in the perpendicular direction, which can be further used to obtain the total energy transferred to the material as $W_{\mathrm{diss}}=vp_{\mathrm{diss}}$ in the nonrecoil approximation [17]. Using (40), this energy may be written as

(41) $$W_{\mathrm{diss}}=v\int_{-\infty }^{\infty }dt\;F_{\mathrm{diss}}\left(t\right)=\int_{0}^{\infty }d\omega \;\left(\hbar \omega \right)\;P\left(\omega \right),$$

where

(42) P(ω)=4Q2πħ∫0∞dk(kv)2ω2+(kv)22Im−1ϵ2D(k,ω).

By the conservation of energy–momentum laws that were demonstrated in [17] under quite general conditions, $W_{\mathrm{diss}}$ must be equal to the energy $W_{\mathrm{loss}}$ lost by the external charged particle as it moves along its trajectory. That energy was evaluated directly as the work of the induced electric field on the external charge, both in the nonrelativistic [37,38] and relativistic [22] regimes. The exact same expression as in (41) and (42) was obtained in [38] for $W_{\mathrm{loss}}$, with P(ω) interpreted as probability density for the incident particle losing the energy ħω, which is typically measured in the STEM-EELS experiments using large collection apertures of the transmitted electron beams [38,39].

## 3. Analytical Expressions and Numerical Computations

### 3.1. Conductivity Models

As discussed earlier, using (14) in (21) and (22) allows us to characterize the problem entirely in terms of a particular choice of the conductivity model. We wish to use a simple enough model that yields an analytical expression for $I_{\mathrm{diss}}\left(k,t\right)$, so that the resulting I(k,t) from (20) can be used along with (19) to perform an inverse Fourier transform pertaining to the convention (1), which only requires a single integration over *k* in (25) and (26) to reveal spatiotemporal dependencies of the axially symmetric induced potential and the induced charge density, respectively.

Relatively simple, yet sufficiently reliable models of conductivity can be invoked for conductive 2D materials in the limit of large wavelengths (small wavenumbers *k*), covering a range of frequencies from the THz to the MIR. The archetypal conductivity model is that of a quasifree 2D electron gas, given by the Drude model

(43) $$\sigma_{\mathrm{Drude}}\left(\omega \right)=\frac{i}{\pi }\frac{D}{\omega +i\gamma },$$

where *D* is the Drude weight and γ is phenomenological damping rate due electron scattering on impurities and lattice defects. In the case of doped graphene, $D=\left(e^{2}/\hbar^{2}\right)E_{F}$, where $E_{F}=\hbar v_{F}k_{F}$ is its Fermi energy with $v_{F}\approx c/300$ being the Fermi speed and $k_{F}=\sqrt{\pi n}$ the Fermi wavenumber for the doping density *n*. In a 2D material with parabolic conduction band, like TMD [25], the Drude weight is given by $D=\pi e^{2}n/m^{*}$, where *n* is the doping density and $m^{*}$ the effective electron mass in that band. The Drude model provides an adequate description of the low-energy intraband electronic transitions in doped graphene in the optical limit, specifically, for the wavenumbers $k\ll k_{F}$ [1,3,33,40].

The applicability of the Drude model in (43) can be expanded to finite wavenumbers *k* by including the effects of Fermi pressure in the 2D electron gas [20]. Unlike the Drude model, the conductivity function with such Fermi correction contains nonlocal effects, i.e., it depends on *k*, and is given by

(44) $$\sigma_{\mathrm{Fermi}}\left(k,\omega \right)=\frac{i}{\pi }\frac{\omega D}{\omega \left(\omega +i\gamma \right)-s^{2}k^{2}},$$

where s>0 is the speed of sound, which in the case of graphene is derived as $s=v_{F}/\sqrt{2}$ [20].

Using the model with Fermi correction (44) in (14) yields an expression for the dielectric permittivity,

(45) $$\epsilon_{\mathrm{Fermi}}\left(k,\omega \right)=1-\frac{2Dk}{\omega \left(\omega +i\gamma \right)-s^{2}k^{2}},$$

which has a gratifyingly correct static limit of a Thomas–Fermi screening model for doped graphene with $\epsilon_{\mathrm{TF}}\left(k\right)=1+k_{\mathrm{TF}}/k$, where $k_{\mathrm{TF}}=2D/s^{2}$ [41]. Note that the model (44) offers natural scales for time, length and speed with characteristic values $t_{c}=s/\left(2D\right)$, $l_{c}=st_{c}$ and $v_{c}=s$, respectively. Solving the equation $\epsilon_{\mathrm{Fermi}}\left(k,\omega \right)=0$ by setting γ=0 in (45) gives a resonant plasma frequency as

(46) $$\omega_{p\mathrm{Fermi}}\left(k\right)=\sqrt{2Dk+s^{2}k^{2}}.$$

While the long-wavelength limit of this frequency exhibits the characteristic square-root dispersion of a 2D free-electron gas, $\omega_{p\mathrm{Fermi}}\approx \sqrt{2Dk}$, its short-wavelength limit is linear, $\omega_{p\mathrm{Fermi}}\approx sk$, which occurs when $k\gg 2D/s^{2}$. It is this acoustic dispersion of the plasmon frequency $\omega_{p\mathrm{Fermi}}\left(k\right)$ that renders the Fermi model (44) difficult to integrate numerically in (25) and (26). Namely, when the loss function in (22) is expressed as Im−1ϵ2D(kω)=π2ωpFermi(k)δ(ω−ωpFermi(k)) in the limit γ=0, the linear dispersion causes massive cancellations of oscillations in the *k*-integral at large *k* values, which occur for distances r<st. That is the main reason we turn attention to another conductivity model, described next.

It was recently shown that the applicability of the Drude model of conductivity (43) can also be expanded to finite *k* vales by adding a correction due to high-energy interband electron transitions, which gives rise to a local form of the improved conductivity model as

(47) $$\sigma \left(\omega \right)=\sigma_{\mathrm{Drude}}\left(\omega \right)-i\alpha \omega ,$$

where α accounts for static screening due to high-energy interband electron transitions in a 2D material [25,28]. This model was found to work particularly well for TMDs at the wavenumbers up to k∼0.4 Å${}^{-1}$[25], while for doped graphene [27,28] and doped phosphorene [29], it improves the agreement with the ab initio data on plasmon dispersion for the wavenumbers $k≲k_{F}$. Using (47) in (14) yields an expression for the dielectric permittivity,

(48) $$\epsilon_{2D}\left(k,\omega \right)=1+ak-\frac{2Dk}{\omega (\omega +i\gamma )},$$

where a=2πα is introduced for simplicity. We note that, for undoped 2D materials with D=0, (48) is reduced to a static dielectric permittivity $\epsilon_{K}\left(k\right)=1+ak$, which arises in the Keldysh model [42] that is often used to calculate the exciton binding energies in 2D semiconductors [43,44]. On the other hand, for doped 2D materials, solving the equation $\epsilon_{2D}\left(k,\omega \right)=0$ by setting γ=0 in (48) gives a plasmon frequency of

(49) $$\omega_{p}\left(k\right)=\sqrt{\frac{2Dk}{1+ak}}.$$

Notice that, at long wavelengths, ak≪1, the Drude term prevails in (47), so that the above expression gives the same square root plasmon dispersion as the Fermi model, $\omega_{p}\left(k\right)\approx \sqrt{2Dk}$, whereas at short wavelengths, ak≫1, the plasmon frequency (49) saturates at constant value (see Figure 3 in [25]), given by

(50) $$\omega_{\infty }=\lim_{k\to \infty }\omega_{p}\left(k\right)=\sqrt{\frac{2D}{a}}.$$

Note that the conductivity model (47) also provides natural scales for time, $t_{c}=1/\omega_{\infty }$, length, $l_{c}=a$, and speed $v_{c}=a\omega_{\infty }=\sqrt{2Da}$. One may estimate that typical values of those parameters in TMDs [25] or in heavily doped graphene correspond to $\hbar \omega_{\infty }∼1$ eV and a∼25 Å [27,28], giving the order of the characteristic scales to be $t_{c}∼1$ fs and $v_{c}∼0.01\times c$. At the same time, while modeling the damping effects in graphene is still an active research topic [45,46], the phenomenological damping rate was found to be quite small in TMDs, ħγ∼1 meV [25], suggesting that the plasmons in those materials may be quite long lived and may therefore propagate over large distances.

We will discuss both the Fermi model (44) and the interband model (47), but devote our attention primarily to the later. For both models, we are able to obtain analytical results for both the dissipative and conservative parts of I(k,t). The inverse spatial Fourier transform must be carried out numerically, however, to obtain results in the spatiotemportal domain, and we have found that the interband model (47) provides a better convergence of the integrals in (25) and (26) when k→∞. Having said that, we note that both conductivity models in (47) and (44) are insufficient at large wavenumbers, on the order of the inverse lattice spacing in a 2D material, so that the results obtained from our numerical integration in (25) and (26) should be taken with some reservations at short distances, r≲1 nm.

Using the permittivity model (48) in (21) gives

(51) $$I_{\mathrm{cons}}\left(k,t\right)=\frac{\pi }{kv}\frac{e^{-kv|t|}}{1+ak}\left[ak+\frac{\omega_{p}^{2}\left(k\right)}{{\left(kv\right)}^{2}+\gamma kv+\omega_{p}^{2}\left(k\right)}\right],$$

whereas using it in (22) gives two different expressions for $I_{\mathrm{diss}}\left(k,t\right)$, depending on whether *k* is greater or less than the critical value

(52) $$k_{c}=\frac{\gamma^{2}}{8D-a\gamma^{2}}.$$

In particular, for $k>k_{c}$ we have

(53) $$\begin{array}{cc} I_{\mathrm{diss}}^{>}\left(k,t\right) & =\frac{\pi }{kv}\frac{e^{-kvt}}{1+ak}\left[\frac{\omega_{p}^{2}\left(k\right)}{{\left(kv\right)}^{2}-\gamma kv+\omega_{p}^{2}\left(k\right)}-\frac{\omega_{p}^{2}\left(k\right)}{{\left(kv\right)}^{2}+\gamma kv+\omega_{p}^{2}\left(k\right)}\right]\\ \; & +\frac{4\pi \omega_{p}^{2}\left(k\right)e^{-\gamma t/2}}{\left(1+ak\right)\Delta^{>}\left(k\right)}\left[\frac{\left({\left(kv\right)}^{2}+\omega_{p}^{2}\left(k\right)-\frac{\gamma^{2}}{2}\right)\sin\left(\frac{t\Delta^{>}\left(k\right)}{2}\right)-\frac{\gamma }{2}\Delta^{>}\left(k\right)\cos\left(\frac{t\Delta^{>}\left(k\right)}{2}\right)}{\left({\left(kv\right)}^{2}-\gamma kv+\omega_{p}^{2}\left(k\right)\right)\left({\left(kv\right)}^{2}+\gamma kv+\omega_{p}^{2}\left(k\right)\right)}\right], \end{array}$$

where

(54) $$\Delta^{>}\left(k\right)=\sqrt{4\omega_{p}^{2}\left(k\right)-\gamma^{2}}.$$

Notice the analogy between $\Delta^{>}\left(k\right)$ and the discriminant for a classical damped harmonic oscillator, which is real when the oscillator is underdamped and becomes imaginary when it is overdamped. For this reason, we identify the $k>k_{c}$ region as underdamped, and the $k<k_{c}$ region as overdamped. We restrict our attention to $\gamma <2\omega_{\infty }$, a reasonable constraint corresponding to taking sufficiently long-lived plasmon modes in a 2D material with large doping density. With this constraint applied to (52), we conclude that $k_{c}>0$, so there will always be an overdamped region for finite rate γ. While the steps to obtain the plasmonic part are slightly different in the overdamped case, we end up obtaining the result we would expect by simply letting $\Delta^{>}\left(k\right)$ become imaginary. So, for $0<k<k_{c}$,

(55) $$\begin{array}{cc} I_{\mathrm{diss}}^{<}\left(k,t\right) & =\frac{\pi }{kv}\frac{e^{-kvt}}{1+ak}\left[\frac{\omega_{p}^{2}\left(k\right)}{{\left(kv\right)}^{2}-\gamma kv+\omega_{p}^{2}\left(k\right)}-\frac{\omega_{p}^{2}\left(k\right)}{{\left(kv\right)}^{2}+\gamma kv+\omega_{p}^{2}\left(k\right)}\right]\\ \; & +\frac{4\pi \omega_{p}^{2}\left(k\right)e^{-\gamma t/2}}{\left(1+ak\right)\Delta^{<}\left(k\right)}\left[\frac{\left({\left(kv\right)}^{2}+\omega_{p}^{2}\left(k\right)-\frac{\gamma^{2}}{2}\right)\sinh\left(\frac{t\Delta^{<}\left(k\right)}{2}\right)-\frac{\gamma }{2}\Delta^{<}\left(k\right)\cosh\left(\frac{t\Delta^{<}\left(k\right)}{2}\right)}{\left({\left(kv\right)}^{2}-\gamma kv+\omega_{p}^{2}\left(k\right)\right)\left({\left(kv\right)}^{2}+\gamma kv+\omega_{p}^{2}\left(k\right)\right)}\right], \end{array}$$

where now

(56) $$\Delta^{<}\left(k\right)=\sqrt{\gamma^{2}-4\omega_{p}^{2}\left(k\right)}.$$

In an anticipation of the numerically obtained inverse Fourier transform to the real spatial dependence via (25), it follows that the plasmon propagation will always have wavefronts that are overdamped at large radial distances *r* from the impact point, which exceed a value proportional to $1/k_{c}=\frac{8D}{\gamma^{2}}-a$. Using the estimated parameters for TMDs [25], this gives distances exceeding 1 cm, while for lightly doped and “dirty” graphene, this may be a much smaller but still a “macroscopic” distance.

We have found that the presence of finite damping rate, which describes dissipative processes due to the electron scattering on the impurities and phonon modes in the material and its surrounding, plays an important role in the dynamics of plasmon excitation. Notice that the expression for $I_{\mathrm{diss}}$ contains terms with the factor $\xi \left(k\right)={\left(kv\right)}^{2}+\omega_{p}^{2}\left(k\right)-\gamma kv$ in the denominator, originating from the pole at −ikv in (18). It is possible to show that this will result in $I_{\mathrm{diss}}\left(k,t\right)$ having an unremovable singularity in its *k*-dependence, which occurs in the overdamped regime, $k<k_{c}$, when we have additionally γ>2D/v. A signature of this singularity may have been observed in [23], where a fully relativistic evaluation revealed that the low-frequency behaviour of the energy loss density P(ω) involves a term $\propto \omega^{-1}\ln\left(2D/\gamma v\right)$ [see Equation (15) with N=1 in [23] for single-layer graphene]. We interpret the value 2D/v as a dynamically-induced upper bound on the maximum possible phenomenological damping rate to avoid the model breaking down. While this is not of a concern for TMDs [25], the complexity of the plasmon damping processes in graphene necessitates further studies [45,46]. In particular, speeds can become quite large in STEM experiments, so the regime γ>2D/v is still of interest for graphene. It is not clear at this stage whether parameters that produce singularities are still physically reasonable, with the inverse Fourier transform possibly producing valid results for such cases if one takes the Cauchy principal value. For now, the large γ, small *v* regime remains in need of further study. Of course, the larger the velocity the more our nonrelativistic approximation breaks down, so we should perhaps not take the surprising conclusion that the maximum permissible damping decreases with increasing velocity too seriously. A relation between the onset of retardation effects at large plasmon propagation distances with the overdamped regime of the plasmon propagation in the nonrelativistic approximation will be further discussed in the Section 3.3 below. At any rate, we avoided this issue in the numerics by restricting the damping by γ<2D/v so that the singularity does not appear in the overdamped regime.

### 3.2. Numerical Results

We use the dimensionless variables $\tau =\omega_{\infty }t$, κ=ak, $\nu =v/v_{c}$, $\Gamma =\gamma /\omega_{\infty }$ to perform computations and to present results.

**Figure 2:**
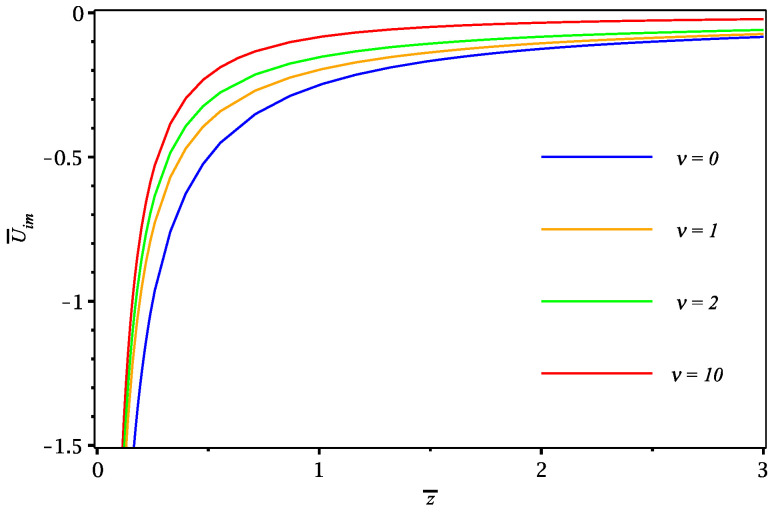
Image potential ${\bar{U}}_{\mathrm{im}}$ in reduced units against the dimensionless perpendicular distance $\bar{z}$ for different reduced speeds ν with damping parameter Γ=0.

We first show in Figure 2 the results for the dynamic image potential in a dimensionless form, ${\bar{U}}_{\mathrm{im}}=\frac{a}{Q^{2}}U_{\mathrm{im}}$, as a function of the reduced perpendicular distance $\bar{z}=z/a$, calculated from the expression

(57) $${\bar{U}}_{\mathrm{im}}\left(\bar{z}\right)=-\frac{1}{2}\int_{0}^{\infty }d\kappa \frac{e^{-2\kappa |\bar{z}|}}{1+\kappa }\left[\kappa +\frac{1}{\nu (1+\kappa )(\kappa \nu +\Gamma )+1}\right],$$

which is obtained from (39) with (51). We remark that the static case with ν=0 (blue curve) in that figure corresponds to the classic image potential, $U_{\mathrm{im}}=-Q^{2}/\left(4|z|\right)$, of a stationary point charge *Q* a distance |z| away from an ideally conducting plane. The case with ν=10 (red curve) is close to the limit of infinite speed, corresponding to the image potential above a 2D semiconductor described by the Keldysh dielectric permittivity $\epsilon_{K}\left(k\right)=1+ak$, giving in normalized form ${\bar{U}}_{\mathrm{im}}\left(\bar{z}\right)=-\frac{1}{4|\bar{z}|}+\frac{1}{2}e^{2|\bar{z}|}\;{Ei}_{1}\left(2\right|\bar{z}\left|\right)$, where ${Ei}_{1}$ is the exponential integral. In that limit, the image potential decreases asymptotically as $U_{\mathrm{im}}∼-Q^{2}a/\left(8z^{2}\right)$.

The singularity in the image potential in Figure 2 at z=0 arises because of the local character of our model conductivity (47). A finite value of the image potential close to a 2D material, which is physically more plausible, can be obtained phenomenologically by introducing a cutoff wavenumber $k_{\mathrm{cut}}$ in the upper limit of the integral in Equation (39). For example, Despoja et al. used $k_{\mathrm{cut}}\approx 15$ nm${}^{-1}$ in [47] to describe the static image potential above graphene.

**Figure 3:**
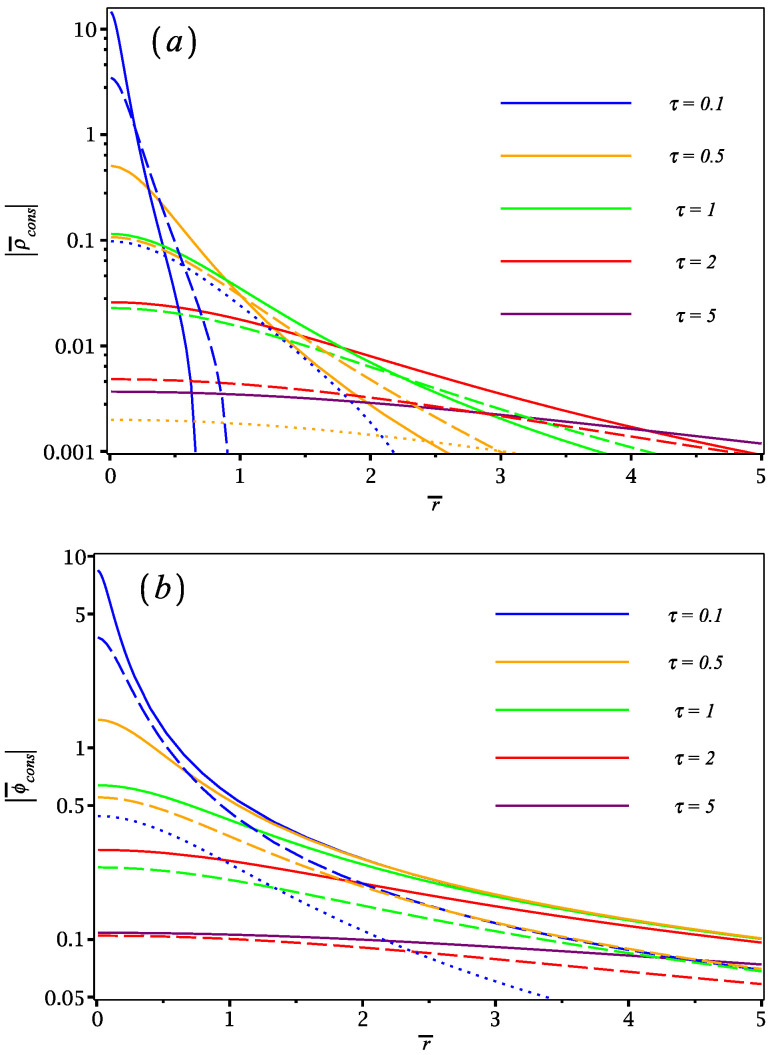
Conservative parts of (**a**) the induced charge density, ${\bar{\rho }}_{\mathrm{cons}}$, and (**b**) the induced potential ${\bar{\phi }}_{\mathrm{cons}}$ in the plane z=0, with their absolute values shown on a logarithmic scale using reduced units against the dimensionless radial distance $\bar{r}$ for different dimensionless times (τ=0.1,0.5,1,2,5), with the damping parameter Γ=0 and the reduced speeds ν=1 (solid lines), ν=2 (dashed lines) and ν=10 (dotted lines).

Using (26) and (25) with (51) where Γ=0, we computed the conservative parts of the induced charge density and the induced potential in the z=0 plane, which are shown in Figure 3 in reduced form, ${\bar{\rho }}_{\mathrm{cons}}=\frac{a^{2}}{Q}\rho_{\mathrm{cons}}$ and ${\bar{\phi }}_{\mathrm{cons}}=\frac{a}{Q}\phi_{\mathrm{cons}}$, as functions of the reduced radial distance $\bar{r}=r/a$ and the reduced time τ at several speeds (ν=1,2,10) for several time points in the interval 0<τ≤5 (recall that ${\bar{\rho }}_{\mathrm{cons}}$ and ${\bar{\phi }}_{\mathrm{cons}}$ are even functions of time). One notices that the induced charge density decreases in magnitude and spreads out radially with increasing time and with increasing speed. Interestingly, its time dependence implies that there exists an approximate scaling with $\bar{z}=\nu \tau$ at radial distances $\bar{r}≲1$. This may be traced to the adiabatic limit of a quasistatic point charge *Q* a distance *z* away from a perfectly conducting plane z=0 [31], giving an upper bound to the curves shown in Figure 3a for each combination of the τ and ν parameters with fixed product ντ. One notices in Figure 3b that the induced potential decreases in magnitude with increasing time and with increasing speed. The radial spread of the curves also decreases with increasing sped but, interestingly, the curves at different times with fixed speed tend to group together at large radial distances.

To carry out the numerical integrals in (25) and (26) using (53) and (55) efficiently is a nontrivial computational problem because of the oscillations in the Bessel function and the trigonometric functions in (53). We were able to adapt numerical methods from [48] based on the Hankel transform to carry out these integrations far more efficiently than was previously possible. This allowed for plots and animations of the dissipative parts of the induced charge density and the induced potential in the *z*-plane, which will be shown in the spatiotemporal domain in a dimensionless form, ${\bar{\rho }}_{\mathrm{diss}}=\frac{a^{2}}{Q}\rho_{\mathrm{diss}}$ and ${\bar{\phi }}_{\mathrm{diss}}=\frac{a}{Q}\phi_{\mathrm{diss}}$, as functions of the reduced radial distance $\bar{r}=r/a$ from the point of impact and the reduced time τ.

**Figure 4:**
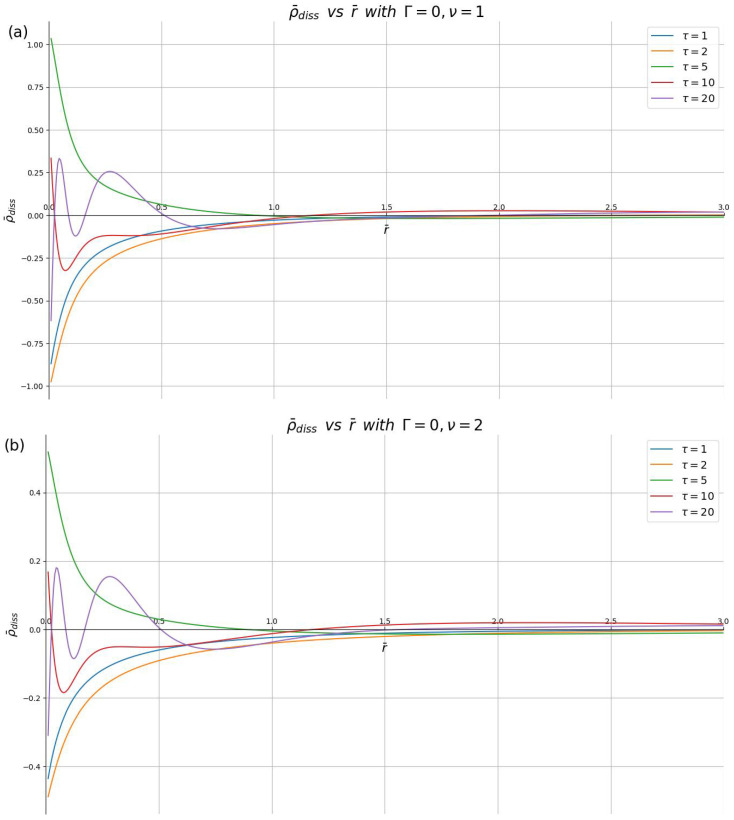

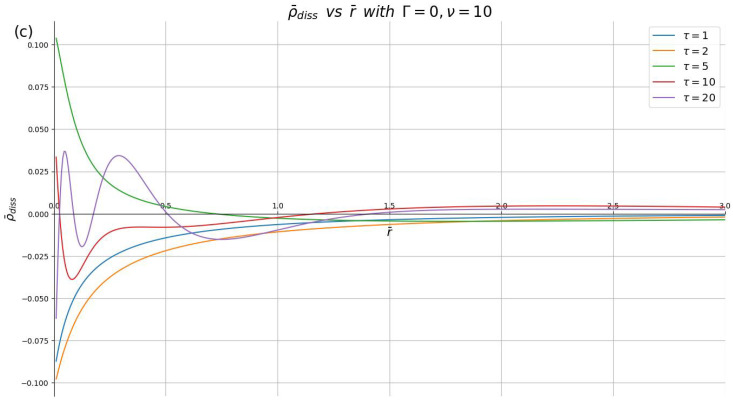
Dissipative part of the induced charge density, ${\bar{\rho }}_{\mathrm{diss}}$, shown in reduced units against the dimensionless radial distance $\bar{r}$ for different dimensionless times (τ=1,2,5,10,20), with the damping parameter Γ=0 and the reduced speeds: (**a**) ν=1, (**b**) ν=2 and (**c**) ν=10.

In Figure 4, we show ${\bar{\rho }}_{\mathrm{diss}}\left(\bar{r},\tau \right)$ at several speeds (ν=1,2,10) for several time points in the interval 0<τ≤20 with the damping parameter fixed at Γ=0. For times τ≲5, one can see a monotonic dependence of ${\bar{\rho }}_{\mathrm{diss}}$ on $\bar{r}$, which changes sign when going from τ=2 to τ=5. At the times τ=10 and τ=20, waves are generated near the origin, so that the charge density ${\bar{\rho }}_{\mathrm{diss}}$ shows one and two wave crests, respectively, located at distances $\bar{r}<1$. The main effect of the increasing speed in Figure 4 is to reduce the overall magnitude of the dissipative charge density, while the structure of the curves (e.g., the positions of the wave crests) and their time evolution are practically unaffected by the speed. In fact, the magnitude of the curves in Figure 4 seems to be scaling almost exactly as ∝1/ν.

**Figure 5:**
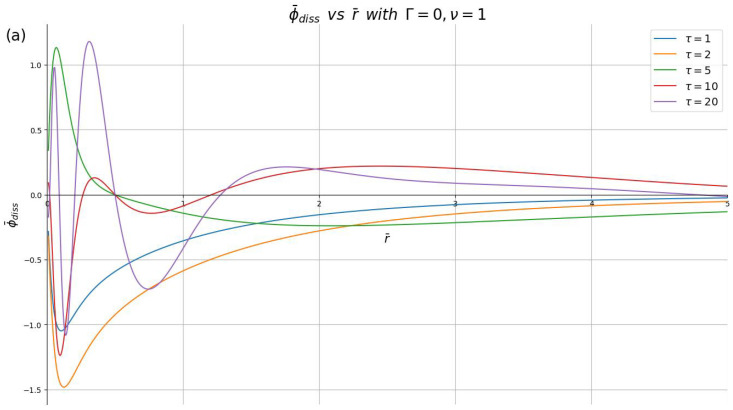

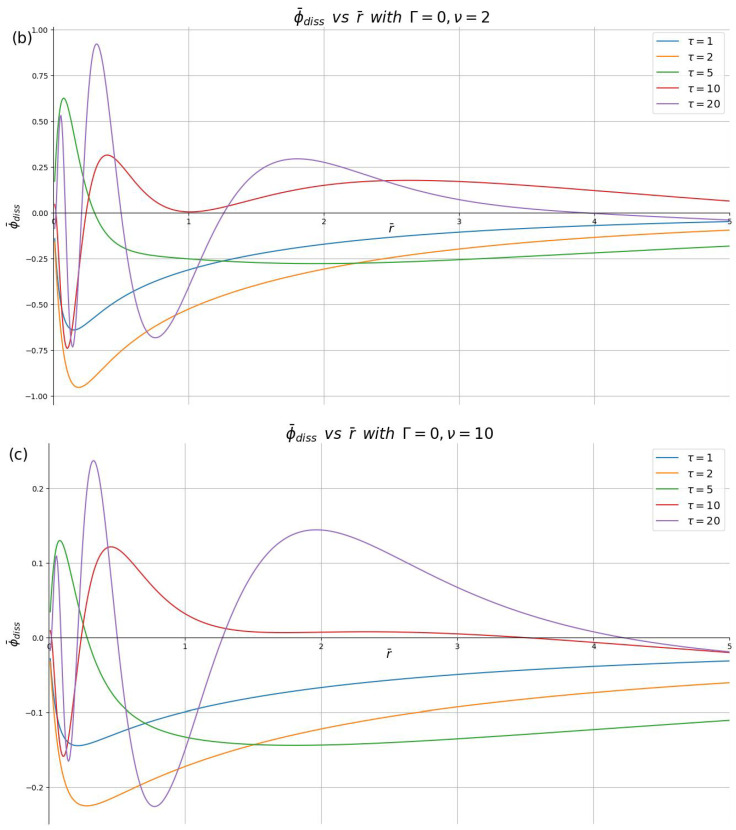
Dissipative part of the induced potential, ${\bar{\phi }}_{\mathrm{diss}}$ in the plane z=0, shown in reduced units against the dimensionless radial distance $\bar{r}$ for different dimensionless times (τ=1,2,5,10,20), with the damping parameter Γ=0 and the reduced speeds: (**a**) ν=1, (**b**) ν=2 and (**c**) ν=10.

In Figure 5, we show ${\bar{\phi }}_{\mathrm{diss}}\left(\bar{r},0,\tau \right)$ using the same parameters as in Figure 4. At the times τ≲5, the potential ${\bar{\phi }}_{\mathrm{diss}}$ exhibits a single extremum at a distance close to the origin, with a change of sign similar to that in the charge density ${\bar{\rho }}_{\mathrm{diss}}$, whereas at the time points τ=10 and τ=20, there are one and two well-developed wave crests in the potential ${\bar{\phi }}_{\mathrm{diss}}$, respectively, which are located at larger radial distances than the corresponding crests in the density ${\bar{\rho }}_{\mathrm{diss}}$. The overall magnitude of ${\bar{\phi }}_{\mathrm{diss}}\left(\bar{r},0,\tau \right)$ decreases with increasing speed, but there is no simple scaling as in the case of the charge density. However, the overall shape of the curves ${\bar{\phi }}_{\mathrm{diss}}\left(\bar{r},0,\tau \right)$ at different time points is preserved, with some modifications, as the speed increases.

**Figure 6:**
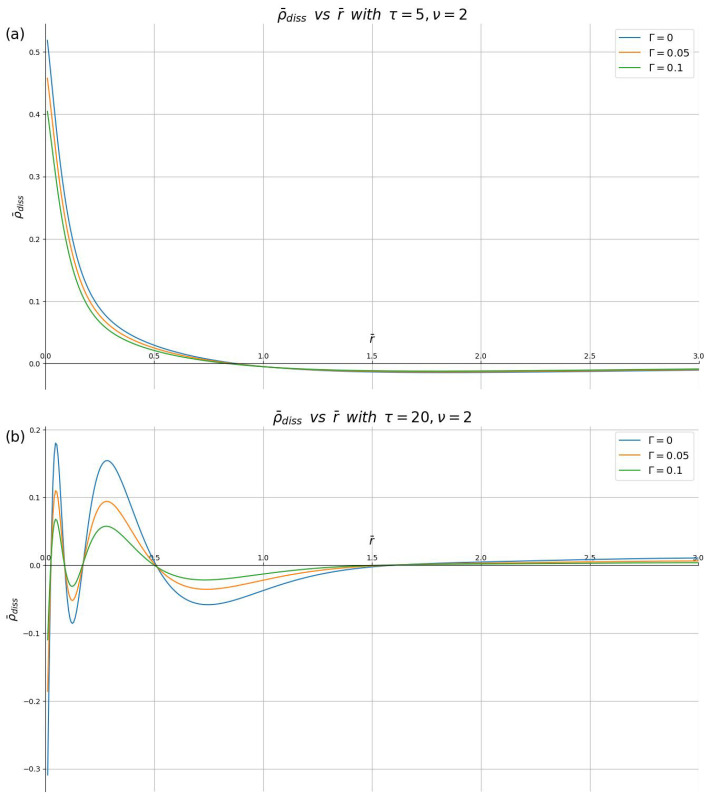
Dissipative part of the induced charge density, ${\bar{\rho }}_{\mathrm{diss}}$, shown in reduced units against the dimensionless radial distance $\bar{r}$ for different damping parameters (Γ=0,0.05,0.1) with the reduced speed ν=2, and the reduced times: (**a**) τ=5 and (**b**) τ=20.

**Figure 7:**
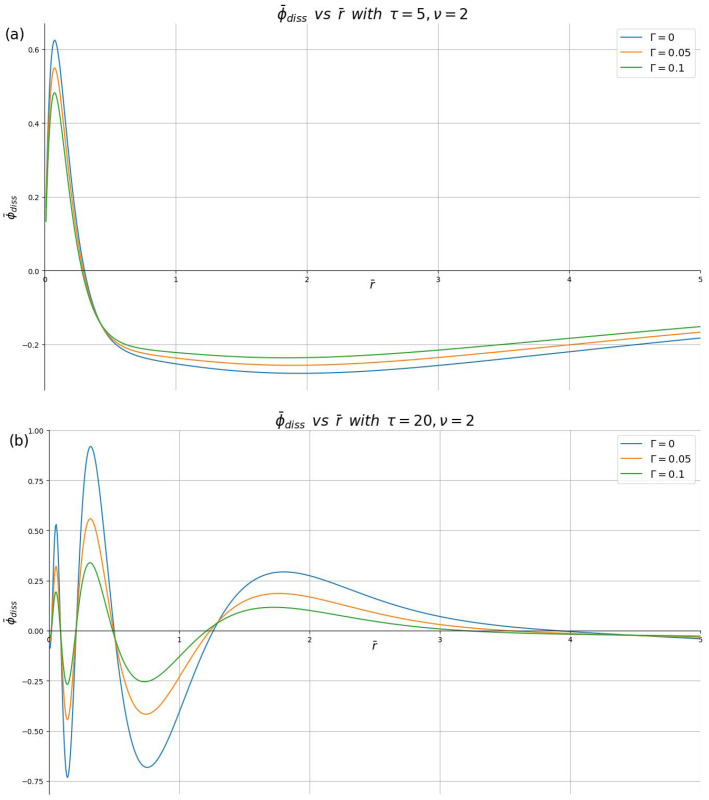
Dissipative part of the induced potential, ${\bar{\phi }}_{\mathrm{diss}}$ in the plane z=0, shown in reduced units against the dimensionless radial distance $\bar{r}$ for different damping parameters (Γ=0,0.05,0.1) with the reduced speed ν=2, and the reduced times: (**a**) τ=5 and (**b**) τ=20.

Finally, in Figure 6 and Figure 7, we show the effects of a finite damping rate on the dissipative parts of the the induced charge density and the induced potential, ${\bar{\phi }}_{\mathrm{diss}}\left(\bar{r},0,\tau \right)$ and ${\bar{\rho }}_{\mathrm{diss}}\left(\bar{r},\tau \right)$, respectively. Having observed in our computations that the relative damping effects are not affected by changing the speed, but that they increase with increasing time, we fix the speed at ν=2 in those two figures, and show results for two representative time points, τ=5 and 20, making sure that we choose Γ<1/ν. One may conclude that the effects of damping are quite small at short times, τ≲5, but they become more pronounced at longer times, with the amplitudes of oscillations in both the density and the potential decreasing, while the overall shape of these oscillations is not changed, as seen for τ=20.

We note that similar oscillations were found in computations of the induced potential using both the analytical Drude model [20] and the ab initio conductivity data for doped graphene [47]. However, both references displayed the total induced potential, $\phi_{\mathrm{ind}}=\phi_{\mathrm{cons}}+\phi_{\mathrm{diss}}$, which exhibits the presence of the conservative part as a transient superimposed on the damped oscillations in the dissipative part of the potential.

### 3.3. Qualitative Analysis Using Stationary Phase Method

Regarding the dynamical evolution of the dissipative part of the induced potential, we note that the dependence of the wave crests on time and on the radial position may be deduced using the method of stationary phase analysis from [21]. Applying the stationarity condition $\nabla_{k}f=0$ on the phase function $f=k\cdot r-\omega_{p}\left(k\right)t$ set to f=−2πℓ gives a pair of parametric functions (with the wavenumber *k* as a parameter) for the radial distance and time,

(58) $$r=2\pi \ell \frac{\omega_{p}^{\prime }\left(k\right)}{\omega_{p}\left(k\right)-k\omega_{p}^{\prime }\left(k\right)},\;\;t=\frac{2\pi \ell }{\omega_{p}\left(k\right)-k\omega_{p}^{\prime }\left(k\right)},$$

which describe the spatiotemporal evolution of the *ℓ*th wave crest in the propagating plasma wave, with $\omega_{p}^{\prime }\left(k\right)=d\omega_{p}\left(k\right)/dk$ being its group velocity.

**Figure 8:**
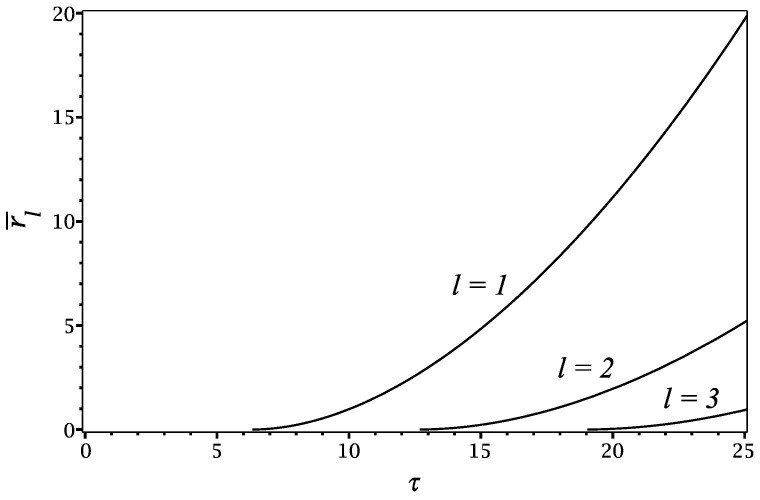
Evolution of the radial position of the *ℓ*th wave crest ${\bar{r}}_{\ell }$ in reduced units as a function of the reduced time τ based on the stationary phase method using the plasmon dispersion (49).

We used the plasmon dispersion (49) to compute the radial positions for ℓ=1,2 and 3, giving of the first, second and third wave crests, which are shown in Figure 8. Those wave crests are created periodically near the origin at r=0 with the period $T=2\pi /\omega_{\infty }$ obtained from (50); they move slowly at short times right after their creation because of the small group velocity at large *k* values, but later, at longer times and large radial distances, the wave crests accelerate according to $r_{\ell }=Dt^{2}/\left(4\pi \ell \right)$ [21] because of the square-root limiting behaviour of the plasmon dispersion (49) at small *k* values. Figure 8 also explains the number of wave crests that can be seen in the dissipative potential in Figure 5 at different time points of its evolution. Except for a single extremum near the origin, there are no fully developed wave crests for times τ<2π, while there is one fully developed and two fully developed wave crests at the time points τ=10 and 20, respectively. This behaviour of the wave crests can be observed in the animations of the dissipative parts of the induced charge density and the induced potential at the reduced speed ν=1 for times τ≤50, which are provided in the video files S1 and S2 in the Supplementary Material, respectively.

As mentioned in the first paragraph of the Section 2.2, an onset of retardation effects may be expected in the plasmon propagation at long distances as a consequence of strong hybridization of the plasmon mode with the light line at long wavelengths, which gives rise to a plasmon polariton mode in doped graphene in the THz range [17,22,24]. A critical wavenumber $k_{\times }$ below which the retardation effects may arise can be estimated from the intercept of the Drude limit of the nonrelativistic plasmon dispersion in (49), $\omega =\sqrt{2Dk}$, and the light line ω=ck (see Figure 1 in [22]). This gives $k_{\times }=2D/c^{2}$ with the corresponding frequency $\omega_{\times }=ck_{\times }$, so that the onset of the retardation effects can be expected when the first wave crest reaches the distance $r_{\mathrm{ret}}∼2\pi /k_{\times }=\pi c^{2}/D$ at the time $t_{\mathrm{ret}}∼2\pi /\omega_{\times }=\pi c/D$. Using the typical parameters for TMDs [25], we can estimate $r_{\mathrm{ret}}∼0.1$ mm and $t_{\mathrm{ret}}∼0.3$ ps, respectively. Because the plasmon polariton dispersion follows the light line when $k\ll k_{\times }$ [22], the acceleration of the wave crests would cease at distanced beyond $r_{\mathrm{ret}}$ and they would continue to move at the speed of light, assuming they could survive the damping by that point.

It is therefore worthwhile comparing the above estimated values of $r_{\mathrm{ret}}$ and $t_{\mathrm{ret}}$ with the distance $r_{\mathrm{damp}}$ and the time $t_{\mathrm{damp}}$ where a transition to the overdamped regime occurs in the nonretarded regime. Based on the discussion in the Section 3.1 and using $k_{c}$ in (52) under the plausible assumption $\gamma \ll \omega_{\infty }$, the first wave crest would experience overdamping when it reaches the distance $r_{\mathrm{damp}}∼2\pi /k_{c}=16\pi D/\gamma^{2}$, with the corresponding time obtained from the above stationary phase analysis as $t_{\mathrm{damp}}∼8\pi /\gamma$. Using the parameters for TMDs with ħγ∼1 meV [25] gives $r_{\mathrm{damp}}∼6.3$ cm and $t_{\mathrm{damp}}∼16.6$ ps, which are far larger than the corresponding values describing the onset of retardation. In general, the ratio of those two time scales is given by $t_{\mathrm{damp}}/t_{\mathrm{ret}}=8\pi \sigma_{\mathrm{stat}}/c$, where $\sigma_{\mathrm{stat}}=D/\left(\pi \gamma \right)$ is the static limit of the Drude conductivity (43), showing a close relation between the retardation and damping in the THz frequency range, i.e., in the picosecond range of the plasmon propagation times. In the case of doped graphene, we have $t_{\mathrm{damp}}/t_{\mathrm{ret}}=8\alpha_{f}E_{F}/\left(\hbar \gamma \right)$, where $\alpha_{f}=e^{2}/\left(\hbar c\right)\approx 1/137$ is the fine structure constant, showing that $t_{\mathrm{damp}}$ may be smaller than $t_{\mathrm{ret}}$ in a lightly doped and/or “dirty” graphene. Finally, using the dynamically induced critical value of the damping parameter from the Section 3.1, $\gamma_{c}=2D/v$, beyond which a singularity occurs in $I_{\mathrm{diss}}^{<}\left(k,t\right)$, the ratio of the two time scales becomes $t_{\mathrm{damp}}/t_{\mathrm{ret}}=4v/c$, showing that the damping is intimately connected with retardation in the overdamped regime, regardless of the parameters *D* and *a* in the conductivity model (47). This relation seems to be a universal property of a 2D conducting sheet at the THz frequencies, which needs to be carefully examined in future work.

As a final comment, the “slowness” of the plasmon modes that emerges at short wavelengths in the conductivity model (47) with the plasmon dispersion (49) was pointed out in [25]. It is manifested in Figure 8 as the quadratic dependence of the *ℓ*th crest’s position, $r_{\ell }=\frac{D}{2\pi \ell }{\left(t-\ell T\right)}^{2}$, for short times right after its creation near the origin, t>ℓT. This is in contrast to the Fermi model of conductivity (44) with the plasmon dispersion (46), where wave crests are generated with the period $T_{\mathrm{Fermi}}=2\pi s/D$ at the circumference r=st of a uniformly expanding circular wavefront that encloses the “calm” region r<st with no waves [21]. Thus, those crests depart from the point of creation with the radial speed that is “inherited” from the acoustic speed *s* of the plasmon mode in the Fermi model, see Figure 4 in [21]. Obviously, the concept of the formation zone and formation time for plasmon launching by an electron impact upon a conductive 2D material greatly depends on the nonlocal effects contained in the model adopted for its conductivity at large wavenumbers *k* [11].

## 4. Concluding Remarks

We have shown how analyzing the problem in the (k,ω) domain allows us to study the spatiotemporal structure of plasmon excitations in a two-dimensional (2D) material penetrated by a charged particle moving on a normal trajectory without recoil. We have developed the theory in the nonrelativistic approximation with an arbitrary in-plane conductivity model that gives rise to an effective 2D permittivity, $\epsilon_{2D}\left(k,\omega \right)$. We described a way to identify conservative and dissipative processes, triggered by the impact of the particle, and we discussed their physical manifestations in terms of the perpendicular force acting on the 2D material, evaluated by means of the Maxwell’s stress tensor. The dynamics of the conservative processes was shown to be fully encoded in the dielectric permittivity as a function of imaginary frequency, $\epsilon_{2D}\left(k,ikv\right)$, whereas their time dependence comes trough the instantaneous distance between the particle and the 2D material, |z|=v|t|. The pertinent force on that material was shown to be the opposite to the dynamic image force on the particle.

On the other hand, the dissipative processes were shown to give rise to the plasmon wave excitations in the 2D material, which account for the total energy and momentum transfer to the material and are fully characterized by its Loss function, Im−1/ϵ2D(k,ω), along the real frequency axis. In other words, the spatiotemporal evolution of the plasmon wave propagation in the 2D material can be evaluated by an integration over the (k,ω) domain, with the Loss function that may be obtained from analytical models, ab initio calculations, or even from the data acquired in a momentum-resolved STEM-EELS experiment, making sure that reliable data are available over the entire (k,ω) domain. We have discussed several analytical models of conductivity, which are regarded as valid in the THz-MIR frequency range and for long wavelengths, but we adopted for numerical computations a model combining the Drude conductivity with the weight *D* and a correction due to the interband electron transitions in the 2D material with an in-plane static polarizability α. The inclusion of finite damping γ in the model acts in a manner analogous to damping in a classical harmonic oscillator, splitting the problem into underdamped and overdamped regimes at short and long wavelengths, respectively. In doing so, we have placed bounds on the permissible values of the damping in relation to the particle speed to avoid model singularities, though the nature of the singularities is not well understood at present. Finally, we have shown how to obtain numerical results for the induced potential and charge density in the 2D material in the spatiotemporal domain, allowing us to visualize the plasmon excitations in the form of animated plots.

We have found that the main effect of the increasing particle speed *v* is to reduce the magnitudes of the dissipative parts of the induced potential and the induced charge density in the 2D material, whereas the overall wave structure and the dynamics of the propagating plasmon are not significantly affected by the particle speed. This observation made it possible to perform a qualitative analysis of the time evolution of the wave crests by the method of stationary phases. Accordingly, the following scenario emerged for the plasmon launching based on the adopted conductivity model: the creation of new wave crests begins at the times t>0 following the impact of the charged particle, it occurs at a constant rate of $\frac{1}{2\pi }\sqrt{D/(\pi \alpha )}$ near the point of impact with the amplitudes of the wave crests that decrease with the increasing particle speed. Following a slow initial departure from the point of creation, the *ℓ*th plasmon wave crest expands to a large radial distance where it propagates with constant acceleration D/(2πℓ) that reflects the square-root plasmon dispersion in the long wavelength limit, which is universal for all quasifree electron gas models of the conductive 2D materials. The plasmon propagates as an underdamped wave to a distance $r_{\mathrm{damp}}∼16\pi D/\gamma^{2}$, beyond which the overdamped regime sets in.

A number of possible directions for future research remain. While the nonrelativistic approximation is expected to work for the particle speeds v≪c, we have also demonstrated that the retardation effects may arise for plasmon propagation at distances exceeding $r_{\mathrm{ret}}∼\pi c^{2}/D$ due to hybridization of the plasmon dispersion with the light line at long wavelengths. Because $r_{\mathrm{ret}}$ can be comparable to $r_{\mathrm{damp}}$, the inclusion of relativistic effects in future work will not only allow us to consider electron speeds of relevance to the (S)TEM observations, but it will also help expose the role of the plasmon damping at the THz frequencies [22,23,24].

Another next step that has already been discussed is the use of existing conductivity data from ab initio calculations as the conductivity model [1,3,33]. Using the expressions already derived for arbitrary conductivity, it is relatively straightforward to extend the numerical methods to invert the temporal Fourier transform numerically as well. This would allow for more realistic results than those obtained from analytical models of conductivity, especially at short wavelengths and large frequencies.

One more topic for future work would include relaxing the no-recoil approximation for low incident electron energies $E_{0}$. While the deflection of an electron beam is not expected to play any significant role in the plasmon launching, the assumption of constant electron speed can be justified if the electron incident energy is such that $E_{0}\gg \left|U_{\mathrm{im}}\right|$. This relation is satisfied for the energies in the low–keV range and higher, after the image potential is corrected to take a finite value near the 2D material. Another problem of practical interest in (S)TEM should consider the electron beam as a succession of single-electron impacts with different arrival times on a 2D target. To be able to apply our linear response theory to studying the dynamics of plasmon launching, it is important that the arrival times are well separated, so that the electron gas in the target can be fully relaxed between two consecutive electron impacts. For the typical electron currents of some 100 pA, the average separation of the arrival times exceeds one nanosecond [15]. While we showed that the typical times that it takes the plasmon wave crests to reach the distance where the overdamping sets in are in the picosecond range, it remains to be examined whether the wave crests generation in the near zone could be fully relaxed within a nanosecond.

Finally, it would also be interesting to evaluate the spatiotemporal dependence of the collective modes excited in an anisotropic 2D material, such as phosphorene [28,29], which exhibits a topological transition from the elliptic to hyperbolic isofrequency plasmon dispersion curves in the $\left(k_{x},k_{y}\right)$ plane. Yet another interesting aspect of the problem involves the effects of finite size of a 2D material target, such as plasmon launching by electron impact upon graphene ribbons [49].

## Data Availability

The data presented in this study are available on request from the corresponding author.

## Supplementary Materials

The following supporting information can be downloaded at: https://www.mdpi.com/article/10.3390/ma16031150/s1, Video S1: Dissipative charge density. Video S2: Dissipative induced potential.
